# Dual processing of visual rotation for bipedal stance control

**DOI:** 10.1113/JP271813

**Published:** 2016-05-27

**Authors:** Brian L. Day, Timothy Muller, Joanna Offord, Irene Di Giulio

**Affiliations:** ^1^ Sobell Department of Motor Neuroscience and Movement DisordersInstitute of NeurologyUniversity College LondonLondonUK

## Abstract

**Key points:**

- When standing, the gain of the body‐movement response to a sinusoidally moving visual scene has been shown to get smaller with faster stimuli, possibly through changes in the apportioning of visual flow to self‐motion or environment motion.
- We investigated whether visual‐flow speed similarly influences the postural response to a discrete, unidirectional rotation of the visual scene in the frontal plane.
- Contrary to expectation, the evoked postural response consisted of two sequential components with opposite relationships to visual motion speed.
- With faster visual rotation the early component became smaller, not through a change in gain but by changes in its temporal structure, while the later component grew larger.
- We propose that the early component arises from the balance control system minimising apparent self‐motion, while the later component stems from the postural system realigning the body with gravity.

**Abstract:**

The source of visual motion is inherently ambiguous such that movement of objects in the environment can evoke self‐motion illusions and postural adjustments. Theoretically, the brain can mitigate this problem by combining visual signals with other types of information. A Bayesian model that achieves this was previously proposed and predicts a decreasing gain of postural response with increasing visual motion speed. Here we test this prediction for discrete, unidirectional, full‐field visual rotations in the frontal plane of standing subjects. The speed (0.75–48 deg s^–1^) and direction of visual rotation was pseudo‐randomly varied and mediolateral responses were measured from displacements of the trunk and horizontal ground reaction forces. The behaviour evoked by this visual rotation was more complex than has hitherto been reported, consisting broadly of two consecutive components with respective latencies of ∼190 ms and >0.7 s. Both components were sensitive to visual rotation speed, but with diametrically opposite relationships. Thus, the early component decreased with faster visual rotation, while the later component increased. Furthermore, the decrease in size of the early component was not achieved by a simple attenuation of gain, but by a change in its temporal structure. We conclude that the two components represent expressions of different motor functions, both pertinent to the control of bipedal stance. We propose that the early response stems from the balance control system attempting to minimise unintended body motion, while the later response arises from the postural control system attempting to align the body with gravity.

## Key points

- When standing, the gain of the body‐movement response to a sinusoidally moving visual scene has been shown to get smaller with faster stimuli, possibly through changes in the apportioning of visual flow to self‐motion or environment motion.
- We investigated whether visual‐flow speed similarly influences the postural response to a discrete, unidirectional rotation of the visual scene in the frontal plane.
- Contrary to expectation, the evoked postural response consisted of two sequential components with opposite relationships to visual motion speed.
- With faster visual rotation the early component became smaller, not through a change in gain but by changes in its temporal structure, while the later component grew larger.
- We propose that the early component arises from the balance control system minimising apparent self‐motion, while the later component stems from the postural system realigning the body with gravity.

## Introduction

The visual system plays a significant role in helping to maintain upright bipedal posture (Edwards, 1946) partly through visual motion providing information about unintended movement of the body. The source of any visual motion, though, is inherently ambiguous – is it due to motion of self or the environment? The brain is susceptible to this ambiguity, as shown originally by Lee and colleagues in their moving‐room experiments. Movement of the room consistently evoked a self‐motion illusion together with a postural response that acted to compensate for the apparent self‐motion (Lishman & Lee, 1973; Lee & Aronson, 1974; Lee & Lishman, 1975). These phenomena are robust and have subsequently been observed many times under different conditions (e.g. Brandt *et al*. 1973; Lestienne *et al*. 1977; Bronstein & Buckwell, 1997). However, not all visual motion is equally ambiguous for the brain. For example, the postural response to a moving visual scene is attenuated when subjects are given precise information about when and how the scene will move in the immediate future (Guerraz *et al*. 2001). Such explicit knowledge of probable causality is not the only information the brain uses to help disambiguate the source of visual motion; properties of the visual motion also play a role. For example, the speed of visual motion has been found to influence the magnitude of the evoked postural response with faster speeds being associated with lower response gains (Peterka & Benolken, 1995; Mergner *et al*. 2005).

Effects of this sort are to be expected if the brain's attribution of causality is determined by an estimate of how much of the visual motion is likely to be due to self‐motion. Dokka *et al*. (2010) put forward a Bayesian model as the basis for this estimation. The model assumes the brain refers to other sensory information that provides alternative noisy estimates of self‐motion, for example vestibular signals, as well as to memories of prior visual experience. For example, past experience would suggest that fast visual motion is more readily attributable to objects moving in the environment than to self‐motion. With such a prior favouring low body speed in the environment, the Bayesian model predicts a decreasing gain of postural response to increasing speed of visual motion, as observed (Peterka & Benolken, 1995; Mergner *et al*. 2005; Dokka *et al*. 2010).

Here we investigate whether a prior for speed of visual motion influences the postural response to discrete and unpredictable visual‐field motion. In previous experiments, continuous sinusoidal stimuli were employed (Peterka & Benolken, 1995; Mergner *et al*. 2005; Dokka *et al*. 2010) and so the measured response represents the steady‐state behaviour to multiple cycles of an intrinsically predictable stimulus. Although oscillatory visual signals can be experienced under natural conditions, for example on a boat, there are many occasions when visual stimuli are discrete and unpredictable, for example a passing vehicle. Is the initial transient postural response to a discrete visual perturbation influenced by visual motion speed in the same way as for oscillatory stimuli? To investigate this we have used visual perturbations consisting of unidirectional, horizontal‐axis rotations of the full visual field in the subject's frontal plane. With the rotation axis at the level of the ankles, the visual flow pattern is compatible with mediolateral body sway. If the scene is made to rotate at speeds greater than those experienced during normal body sway, the likelihood of it being due to self‐motion is reduced. For these higher visual motion speeds, therefore, a Bayesian model (Dokka *et al*. 2010) would predict a negative relationship between stimulus speed and the size of the evoked postural response. However, we find that the behaviour evoked by this discrete visual rotation is richer than expected, requiring a more complex interpretation.

## Methods

### Ethical approval

Procedures were approved by The National Research Ethics Service Committee (London – Central). Participants gave written, informed consent to the experiment, which conformed to the *Declaration of Helsinki*.

### Subjects

Nineteen subjects (8 male, 11 female) with a mean age of 26.6 years (range: 20–37) and a mean height of 1.76 m (range: 1.60–1.97) consented to take part. Ten subjects participated in the first experiment and 11 in the second experiment, with 2 subjects participating in both. All were healthy individuals with no current or past injury or illness that could affect balance. All subjects’ eyesight was normal or corrected to normal.

### Experimental setup

A 2.4 m wide rear‐projection screen (The Widescreen Centre Ltd, London, UK) was suspended from floor to ceiling 0.55 m in front of a force plate (model 9281B, Kistler, Winterthur, Switzerland). Subjects stood without shoes in the middle of the force plate and faced the screen. Their toes were placed 0.6 m in front of the screen so that their eyes were approximately 0.7 m from the screen. Infrared‐emitting diodes (IRED) were taped to the skin overlying the C7 spinous process, T7 spinous process, and the left and right posterior superior iliac spine. The three‐dimensional (3‐D) motion of the markers was recorded (Coda, Charnwood Dynamics, Rothley, UK) together with the 3‐D forces and moments from the force plate at a sampling frequency of 200 Hz.

Visual stimuli were generated in MATLAB (Mathworks, Natick, MA, USA) using the Psychophysics Toolbox (Brainard, 1997; Pelli, 1997; Kleiner *et al*. 2007). A central control computer communicated with both the data collection computer and the visual stimulus computer. This ensured that the signal to instruct movement of the visual scene was time‐locked to the signal to start data collection. However, there was a variable delay between the instruction to start visual scene movement and actual movement of the scene. This delay was measured using the same three interacting computers and software together with a photodiode (model 10530DAL; IPL, Dorchester, UK) attached to a screen driven by the visual stimuli computer. The photodiode output was captured by the data collection computer at a sampling frequency of 1 kHz. In 96 trials the measured delay had a mean value of 30.8 ms and a standard deviation of 8.9 ms. Unless otherwise stated, all measurement times and temporal values in the figures are taken from the time of the signal to start the visual scene movement rather than the time of actual scene movement, which could not be measured during the experiments.

Visual stimuli were rear‐projected onto the screen. The visual scene had a width of 2.4 m and a height of 2.0 m. It consisted of 18 mm diameter circular dots of various colours (white, red, dark blue, light blue, dark green, light green, pink, yellow, purple) randomly distributed over the screen at a uniform density of 300 dots m^–2^ on a black background. The visual scene was set to rotate in the subject's frontal plane about a horizontal axis at ankle height in the subject's mid‐line, as this would be the axis of rotation of the external world on the retina resulting from mediolateral body sway.

### Experimental protocol

Subjects were asked to stand relaxed with feet together, hands by their side and to look straight ahead at the visual scene. They were told to look at the scene in whichever way felt natural to them without moving their head. Lights were turned off making the visual scene the only light source available to the subject, and the room was quiet and no talking permitted to eliminate auditory reference cues to location in space. Without the subject's knowledge and at irregular intervals, an experimenter pressed a button which initiated a trial after a random delay (0.5–1.5 s).

#### Experiment 1

There were 15 experimental conditions consisting of a static condition, in which the dots did not move, and 14 visual‐motion conditions made up of seven different angular velocity profiles presented clockwise and anticlockwise. Visual‐motion stimuli started with a 0.5 s velocity ramp, during which time the visual scene went at constant angular acceleration from zero velocity to the angular velocity of that trial. There were then 4 s of constant angular velocity rotation followed by another 0.5 s ramp as the scene decelerated back down to zero velocity, totalling 5 s of stimulation per trial. Each trial consisted of 10 s of data collection with the visual stimulus starting at 2 s. The constant angular velocities employed were incremented logarithmically from 0.75 to 48 deg s^–1^ (0.75, 1.5, 3, 6, 12, 24 and 48).

There were 20 trials per angular velocity per subject, 10 clockwise and 10 anticlockwise, and 20 static trials in which the dots remained stationary. Trials were pseudo‐randomly delivered in five blocks of 32, giving 160 trials per subject. Each block lasted approximately 8–9 min.

#### Experiment 2

There were 7 experimental conditions consisting of a static condition and 6 visual‐motion conditions made up of three different angular velocity profiles presented clockwise and anticlockwise. The three profiles were: (A) constant angular velocity at 12 deg s^–1^ for 6 s starting from zero velocity (effectively infinite acceleration at the start, which is possible with computer‐generated images); (B) constant angular acceleration at 4 deg s^–2^ for 3 s starting from zero velocity, followed by constant angular velocity at 12 deg s^–1^ for 3 s; and (C) constant angular acceleration at 4 deg s^–2^ for 6 s starting from zero velocity. These values were chosen on the basis of the robustness of responses obtained in experiment 1.

Unlike in experiment 1, the visual scene was obscured before and after the stimulation period using liquid crystal goggles (PLATO, Translucent Technologies, Toronto, Canada). This was to prevent anchoring of gaze on specific dots during the pre‐stimulus period. Thus, trials began with vision being occluded for 3 s. The goggles were then made clear for 6 s while the visual motion stimulus was applied, after which vision was occluded again for 3 s. Subjects underwent 80 trials of 12 s duration consisting of 20 static trials, in which the dots remained stationary, and 10 trials of each visual‐motion condition. These were administered pseudo‐randomly in five blocks of 16 trials each. Each block lasted approximately 6–7 min.

For both experiments, subjects took a few minutes break between blocks to prevent fatigue, and the room lights were switched back on to prevent significant dark adaptation which accelerates after approximately 10 min and takes around 30 min (Hecht *et al*. 1937; Lamb 1981). This minimised the availability of peripheral visual information of the static surroundings from the little light in the room.

### Data analysis

The data were analysed off‐line using MATLAB. To reduce noise, marker velocity data were low‐pass filtered using a third order, zero‐phase lag Butterworth filter with a 5 Hz cut‐off frequency.

Stimulus‐aligned trials from each condition/subject were initially averaged (note that trials were aligned to the signal to start visual motion; see experimental setup for details). In addition, to extract a single response for each stimulation velocity, data were combined for clockwise and anticlockwise stimuli. To achieve this, the mediolateral components of the kinematic and kinetic responses to anticlockwise stimuli were first multiplied by −1 before averaging with their clockwise counterparts. Grand mean traces were then obtained by averaging across subjects for each condition or each stimulus velocity when directions were combined. During the response, the body approximated an inverted pendulum such that all markers and centre of pressure showed very similar patterns of displacement (data not shown). We have therefore restricted our kinematic analyses to the marker at C7, which gave the largest response magnitude as it was at the top of the body.

The upper limit of each subject's spontaneous body sway speed was estimated from mediolateral velocity of the C7 marker during static trials. The fluctuations of body velocity about its zero mean value gave the required information. We calculated 1.96 × standard deviation of velocity fluctuations as a cut‐off value since for a normal distribution the body would exceed this velocity magnitude only 5% of the time. This value was obtained for each of the 20 static trials and the mean value calculated for each subject. Finally, the spread of these values across the group as a whole was calculated as the group mean ±1.96 × group SD.

The kinematic and kinetic data were measured at specific times relative to stimulus onset for each subject separately. Statistical analyses were performed using SPSS (IBM SPSS 20, Armonk, NY, USA). One‐factor or two‐factor (where appropriate) ANOVA with repeated measures using the GLM procedure was used to test the null hypothesis that visual rotation velocity profile had no effect on the measured variable. Mauchly's test of sphericity was applied to the data and when significant the degrees of freedom were adjusted using Huyn‐Feldt correction. Alpha was set at 0.05 for significance.

## Results

**Figure 1:**
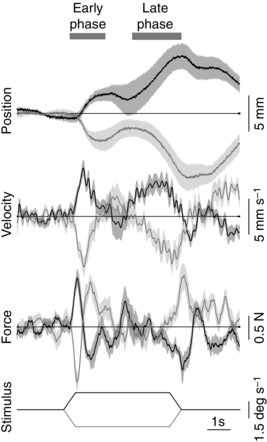
**Example group mean responses** Mean responses (*n* = 10) (±SEM indicated by shaded areas) to one visual scene velocity profile (plateau speed 1.5 deg s^–1^) presented in clockwise (black traces) or anticlockwise (grey traces) directions. Traces from bottom: visual scene velocity profile, mediolateral ground reaction shear force on the body, mediolateral velocity and position of the body at the level of C7. Positive deflection denotes clockwise for stimulus traces and rightwards for position, velocity and force traces. Note the symmetrical behaviour in the two directions and the two response phases marked by horizontal bars at top.

All subjects responded by moving their body laterally in the direction of the visual stimulus. Figure 1 shows this for one stimulus speed in the two directions for experiment 1. These traces also illustrate the time‐varying nature of the response. In general, the response consisted of two phases that were evident in the position and velocity traces during the ramp‐up and constant velocity parts of the stimulus. We refer to these as the early and the late phases. A similar sequence occurred in the opposite direction at the end of the stimulus in response to the ramp‐down to zero velocity.

**Figure 2:**
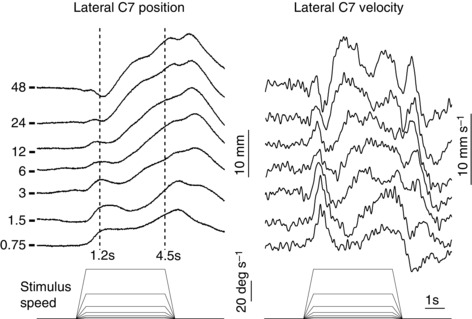
**Effect of visual scene motion profile on kinematic responses** Group mean (*n* = 10) mediolateral displacement (left) and velocity (right) of the body at the level of C7 to different visual scene velocity profiles shown at bottom. Responses to clockwise and anticlockwise stimuli have been combined and averaged. Positive deflection indicates towards the direction of the stimulus. The plateau velocity magnitudes associated with each response are indicated on the left of position traces. Short horizontal lines on the left show the baseline level for each position trace. Vertical dashed lines indicate the measurement times.

Figure 2 shows the differential effect of stimulus speed on the two response phases. At the lowest speed (0.75 deg s^–1^), the two phases produced similar displacements of the body, but the late‐phase displacement developed slower than the early‐phase, and with an apparent pause of the order of 1 s between the two. With faster stimulus speeds the response pattern changed in a number of ways. The early‐phase displacement became smaller, but with little change in latency, while the late‐phase displacement became larger and faster and started earlier. At the fastest stimulus speed (48 deg s^–1^), the early phase developed into a response that moved subjects in the opposite direction to the visual scene direction. This early movement was rapidly reversed by a vigorous late‐phase response.

**Figure 3:**
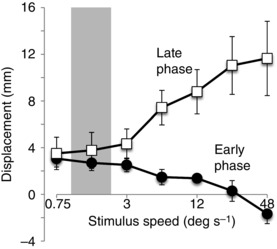
**Effect of visual scene plateau velocity magnitude on body displacement** Group mean (±SEM) mediolateral displacement at the level of C7 shown separately for the early‐phase (filled circles) and late‐phase (open squares) responses plotted against visual scene plateau velocity on a logarithmic scale. The shaded area denotes the mean ± 1.96 × SD of upper‐limit spontaneous sway velocities for the group recorded during static trials.

The differential effect of stimulus speed on the two response phases was measured from C7 lateral displacement in the direction of visual‐field motion at two time‐points (1.2 and 4.5 s after stimulus onset; vertical dashed lines in Fig. 2). The early‐phase response was estimated from C7 position at the first time‐point relative to mean baseline (during the 3 s period before stimulus onset) and the late‐phase response from the difference in position between the two time‐points (1.2 and 4.5 s). The effects of stimulus speed on these two measures are shown in Figure 3. Inspection of this graph suggests that visual motion speed influenced the amplitudes of the two response phases, particularly when stimulus speed exceeded the upper range of normal mediolateral sway velocities measured during static trials (shaded area in Fig. 3). A significant phase × speed interaction (*F*_2.46,22.14_ = 8.051, *P* = 0.001) showed that the speed–amplitude relationships for the two response phases were different from each other. To understand this interaction, the effect of speed on displacement amplitude was analysed for each phase separately. For the early phase there was a significant effect (*F*_3.85,34.68_ = 6.274, *P* = 0.001) indicating a decreasing displacement with speed. For the late phase there was also a significant effect (*F*_1.91,17.21_ = 4.816, *P* = 0.023), but this time indicating an increasing displacement with speed. Thus, the speed–amplitude relationships were in opposite directions for the two response phases.

**Figure 4:**
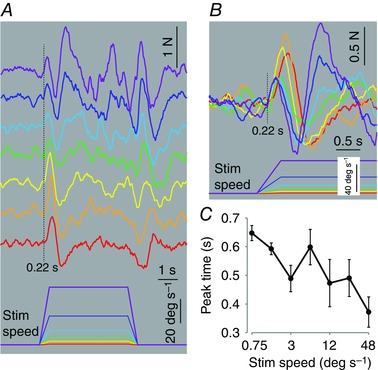
**Effect of visual scene profile on force response** *A*, group mean (*n* = 10) mediolateral ground reaction force after averaging responses from clockwise and anticlockwise stimuli. Positive deflection indicates towards the direction of the stimulus. Visual scene velocity profile (bottom) and the associated responses are colour coded for identification. Order of traces same as for Fig. 2. *B*, same data as *A* but with time scale expanded and traces superimposed with same colour coding. *C*, group mean (±SEM) time of initial peak force response plotted against visual scene plateau velocity on a logarithmic scale.

Inspection of the ground reaction forces showed that the early‐phase response consisted of a sideways force that accelerated the body in the direction of scene motion followed by a decelerating force in the opposite direction (Fig. 4 *A*). After taking account of the mean delay of 30 ms between the signal to move the dots and actual dot movement (see Methods), the onset latency was around 190 ms (220–30 ms) and was relatively independent of stimulus speed (Fig. 4 *B*). The rates of force increase and decrease were also independent of stimulus speed, as shown by relatively fixed positive and negative slopes of the initial force profile (Fig. 4 *B*). However, stimulus speed did affect the time the decelerating force pulse was applied. This altered the time the initial pulse of force peaked (*F*_5.83,52.48_ = 3.071, *P* = 0.013), occurring earlier with greater stimulus speed (Fig. 4 *C*). This temporal shift of the decelerating force explains the decrement of the early‐phase body displacement with greater stimulus speed, and the negative displacement (opposite direction to scene motion) at the fastest speed (Fig. 3). The response onset always occurred during the ramp phase of the stimulus profile, as did the reversal most of the time. This suggests that visual motion acceleration was probably the key variable determining these phenomena.

The early forces were followed by a second more diffuse force pulse acting in the direction of scene motion (Fig. 4 *B*) and which initiated the late‐phase body displacement. In contrast to the early pulse, its latency was less fixed with a tendency to start earlier as stimulus speed increased. Its force profile was also variable and tended to develop faster and reach greater peak amplitude with increasing stimulus speed.

### Relationship between response phases and stimulus structure

The appearance of two response phases may have been a direct result of the stimulus structure, such that the constant acceleration segment evoked the early phase while the constant velocity segment evoked the late phase. To investigate this we manipulated the stimulus structure in a second experiment. Three visual scene rotation profiles were used consisting of (A) constant velocity throughout, (B) constant acceleration for 3 s followed by constant velocity for 3 s and (C) constant acceleration throughout. If the onset of constant velocity stimulation were the trigger for the late‐phase response we would expect it to appear soon after stimulus onset for profile A, to appear after 3 s for profile B and to be absent for profile C.

**Figure 5:**
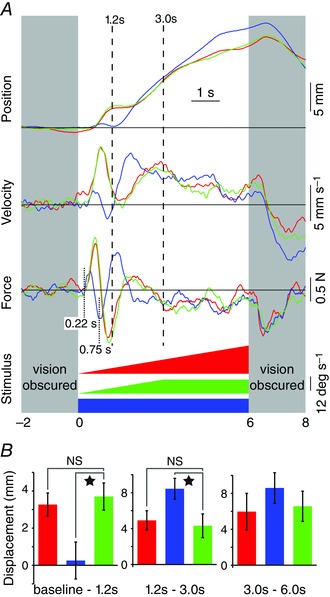
**Effect of visual scene velocity profiles used in experiment 2 on kinematic and kinetic responses** *A*, group mean (*n* = 11) mediolateral responses colour coded according to visual scene velocity profiles (bottom). From top, mediolateral body displacement and velocity at the level of C7 and ground reaction force. Responses averaged for clockwise and anticlockwise stimuli. Positive deflection indicates towards the direction of the stimulus. Grey areas denote periods when subjects were deprived of vision using liquid crystal goggles. Vertical dashed lines indicate measurement times. *B*, group mean (±SEM) C7 displacement measured over the three intervals of baseline to 1.2 s, 1.2–3.0 s and 3.0–6.0 s (panels left to right, respectively). Bars colour coded according to the stimulus profile shown in *A*. When ANOVA indicated a significant effect of stimulus profile on displacement magnitude the data were investigated using analysis of contrasts with the ramp and hold profile (green bar) as the reference condition. Star indicates a significant difference (*P* < 0.05). NS, not significant.

The results did not conform to these predictions. Profiles B and C (green and red traces in Fig. 5) evoked almost identical mean responses (Fig. 5 *A*), which were not significantly different when measured over three separate intervals (baseline to 1.2 s, 1.2–3.0 s, 3.0–6.0 s; Fig. 5 *B*). The response to profiles B and C consisted of a robust early phase followed by a late phase that started during the constant acceleration segment of the stimulus. The late phase was therefore not contingent upon the stimulus change from constant acceleration to constant velocity. Profile A (blue traces in Fig. 5), which consisted of a constant velocity stimulus throughout, evoked a significantly smaller early‐phase than the other profiles (Fig. 5 *B*, left panel), but a more vigorous late‐phase response (Fig. 5 *B*, middle panel). As for experiment 1, the attenuation of the early‐phase response was achieved through a rapid reversal of the initial force response (Fig. 5 *A*). Note that the small early‐phase response to the constant velocity profile is consistent with visual scene acceleration (quasi‐infinite in this case) being the determining factor, as suggested by experiment 1. However, during this early phase, the body did not travel consistently in the opposite direction to visual motion, unlike the response to the highest acceleration in experiment 1. This could have been because the late‐phase response latency occurred earlier to a velocity profile with quasi‐infinite acceleration than with the lower acceleration stimuli of experiment 1. This is consistent with the reduction in late‐phase latency to stimuli with higher velocity, hence higher acceleration, noted in experiment 1. With a latency of around 0.75 s (including the 30 ms mean delay for dot movement onset), the late‐phase onset would have interrupted the deceleration pulse of the early‐phase response (event shown by dotted line at 0.75 s in Fig. 5) and thereby arrested the negatively directed body sway. There was no significant effect of stimulus profile on displacements measured from 3 to 6 s (Fig. 5 *B*, right panel).

## Discussion

As expected, based on the results of many previous studies (see Introduction), we found that movement of the visual environment induced movement of the standing body in the same direction as scene motion. Our initial aim was to examine whether the amplitude of this postural response to a discrete, full‐field rotation in the frontal plane would show a negative relationship with visual‐motion speed, as observed for sinusoidal stimuli (Peterka & Benolken, 1995; Mergner *et al*. 2005; Dokka *et al*. 2010). However, our results revealed some hitherto unreported findings that precluded a simple interpretation. First, the postural behaviour induced by the stimulus was complex, consisting broadly of two components. Second, the amplitudes of the two components were indeed sensitive to visual‐motion speed, but with diametrically opposite relationships. Third, the speed‐dependent size variation of the component that did show the predicted negative relationship was not achieved by conventional gain adjustments.

To our knowledge, the co‐occurrence of two components of postural response to visual motion stimuli has not been reported previously, although the components’ latencies of around 190 ms and >0.7 s do have some correspondence with previous findings. Some groups have reported postural response latencies of more than 1 s (Lestienne *et al*. 1977; Previc & Mullen, 1990) whereas others have reported much shorter latencies of around 250–300 ms (Bronstein & Buckwell, 1997; Guerraz *et al*. 2001). This could reflect variable manifestations of our two postural components, but it is not clear why different experimental approaches should favour one component over the other. Even more fundamental is why our relatively simple stimulus of visual rotation should evoke two components of postural response at all. To address this, the subsequent discussion will focus on what the physiological functions of the two components might be.

### Are both responses corrections for apparent self‐motion?

A commonly held view is that the function of a postural response to visual flow is to correct for unplanned body movement. This requires that a central process attributes at least part of the visual motion to self‐motion, a concept that is supported by the perceptual illusion of vection evoked by the same visual‐flow stimuli. Typically, vection takes some seconds to appear after stimulus onset (Brandt *et al*. 1973; Previc & Mullen, 1990) and therefore starts later than the postural response. This longer latency has been explained on the basis that both arise from the same fundamental process, but with vection having a higher threshold (Previc & Mullen, 1990; Tanahashi *et al*. 2007). Although a postural response can occur without perception of visual motion (Stoffregen, 1986) or vection (Tanahashi *et al*. 2007), it is larger when vection is present (Thurrell & Bronstein, 2002; Tanahashi *et al*. 2007). Therefore, self‐motion illusions and postural responses to visual flow appear to be linked, thus supporting the idea that the postural response serves as a correction for apparent self‐motion. However, this argument is difficult to maintain for both of the components of postural response observed here.

Consider first the sequential pattern of ground reaction forces during just the early response component. The initial event was a self‐generated force acting on the body to accelerate it in the direction of visual motion. Shortly afterwards, a self‐generated force in the opposite direction was recorded that either arrested or reversed the initial body displacement. These motor outputs may reflect sequential decisions about the extent to which visual motion is deemed to be due to environment or self‐motion. The implication is that initially a process attributed part or all of the visual motion to self‐motion, but then on accumulation of further evidence reversed this interpretation, attributing the visual flow more to environment motion. A problem occurs when we try to invoke the same process to the next stage in the sequence, namely the late response component that moved the body again in the direction of visual motion. Why should the process again attribute visual motion to self‐motion? What further evidence could have arisen that would lead to this second reversal of attribution? It seems more likely that the two response components do not stem from the same self‐motion correction process. Instead they may arise from separate processes with different functions, but both of which are pertinent to the control of upright stance.

### The role of the late response in postural alignment with gravity

Dichgans *et al*. (1972) provided strong evidence that a rotating visual scene profoundly influences the brain's estimate of the direction of gravity. First they showed that rotation of a random dot pattern in the frontal plane caused subjects to tilt a straight edge that they were attempting to keep vertical manually. The degree of tilt increased with the angular velocity of the dots, saturating on average at 15 deg  with angular velocities of around 30 deg s^–1^. They went on to show that an equivalent rotating visual motion, using stripes presented to peripheral vision of subjects seated inside a flight trainer, caused the subjects to tilt the trainer in the direction of visual motion. Stripe velocities of 14–26 deg s^–1^ caused an average tilt of 8.5 deg. These experiments therefore demonstrated that rotating visual scenes not only distort the brain's estimate of the direction of gravity but also that this estimate is used to orientate the body in space. This phenomenon could underlie part of the postural response to visual scene rotation that we observed. Of the two postural components that we identified, the late‐phase component shares key characteristics with gravitational tilt to a rotating visual field. Both were found to increase as a non‐linear function of angular velocity, with saturation occurring in the region of 30 deg s^–1^.

The direction of gravity is important for the control of upright stance since the body has to be approximately aligned with the gravity vector when on stationary ground. If the gravity vector is misrepresented in the brain then the body will tend to lean off‐vertical. However, if the rotating scene causes distortion of the gravity vector, why in the present experiments did subjects not simply lean to a new angle and stay there? Why did they continue to move until the scene movement ceased? The answer to this can be found in the slow dynamics of the distortion of verticality. Dichgans *et al*. (1972) showed that the apparent tilt of gravity and tilt of spatial orientation took on average around 17–18 s to reach a steady state. Thus, the gravity distortion would have been slowly increasing during the relatively short rotation periods (5 and 6 s) used in the present study, leading to the observed continuing lean of the body. With this interpretation, the late response would be the component of the postural adjustment responsible for aligning the body with gravity.

### The role of the early response in balancing the body

To stand upright and still requires not only an approximate alignment of the body with gravity, but also minimisation of body sway about this set point. Body sway is minimised by the balance control system, which detects unintended self‐motion and quickly acts on it. Our early postural response to visual rotation satisfies these requirements. The response was initiated quickly (∼190 ms) and had a capability to disambiguate environment motion from self‐motion. The response had a structure reminiscent of a bang–bang controller. Thus, the initial force acting on the body in the direction of visual scene motion had a constant latency and rate of force development for all stimulus profiles. Similarly, the subsequent decelerating force acting in the opposite direction also had a constant rate of force development. Modulation of the response was achieved by altering the interval between these two fixed events. With greater visual scene acceleration, this interval was reduced leading to smaller body displacements. This structure ensures a rapid response onset, but with scope for modification by information processed later.

Clearly this later information stems from properties of the visual scene motion since modification of the early response occurred progressively earlier with more vigorous visual motion. What type of process could this be? One possibility is that it involves the assessment of the likelihood of the visual motion arising from environment motion. This could come from short‐ or long‐term prior experience, as suggested by the major effects occurring when visual rotation speeds exceeded those most commonly experienced during quiet stance (indicated by the shaded bar in Fig. 3). It could also come from explicit knowledge of environment motion or from comparison with other sensory signals. For the latter case, vestibular and somatosensory signals provide independent self‐motion information and so could be used as reference sources. In general, a Bayesian integration process could be implemented to estimate this likelihood, and has indeed been demonstrated to account for both multisensory integration and combining sensory information with prior experience to estimate the most likely true cause (Kording & Wolpert, 2004; Dokka *et al*. 2010; Jazayeri & Shadlen, 2010; O'Reilly *et al*. 2012). Whatever the process, it would require an accumulation of evidence to reach a decision on the source of the visual motion, which would explain the longer time required for processing compared to just detecting and responding to motion. The accumulation of evidence would be quicker for more vigorous visual stimuli, which would explain the observed shorter modification time for faster visual motion stimuli.

### Neural processes

**Figure 6:**
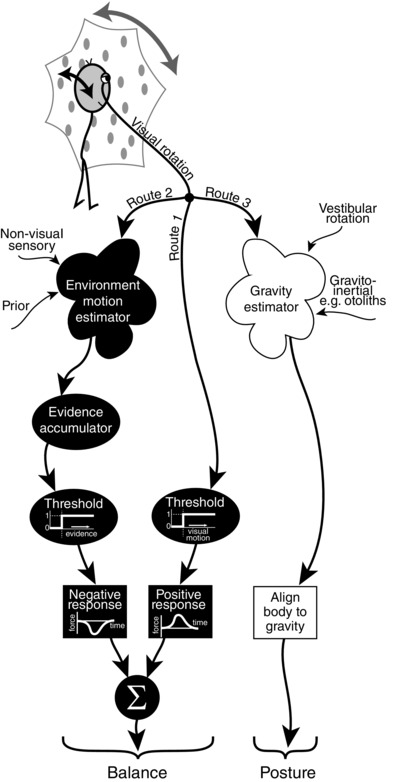
**Schematic representation of hypothetical flow of visual information through parallel sensorimotor processes to produce observed behaviour** Relative motion of self and environment gives visual rotation signal that is fed through three parallel pathways. Route 1 is the fastest passing simply through a visual motion threshold process to evoke a positive response that accelerates the body in the direction of visual rotation. Route 2 is slower as visual rotation is processed together with non‐visual sensory signals and prior information to estimate the likely contributions of environment and self‐motion. If accumulation of evidence for environment motion passes through a threshold then a negative response is evoked which accelerates the body in the opposite direction to visual rotation. These two routes aid the balance system by rapidly identifying and minimising unintended body motion in a bang–bang controller fashion. Route 3 is the slowest with visual rotation being processed together with vestibular rotation, i.e. from semicircular canals, to disambiguate the gravito‐inertial sensory signals, for example from otoliths, to arrive at an estimate of gravity direction. This route aids the postural system by providing a gravitational reference for aligning the body.

A summary of our proposed hypothesis to explain the results is shown diagrammatically in Figure 6. Presumably both proposed processes, dynamic balance and postural alignment, utilise the same raw visual information, but it is plausible that they engage separate brain networks. Indeed, visual flow information is transmitted from retina to a number of cortical and subcortical structures via parallel pathways (Frost *et al*. 1990). The balance system responsible for the early component of the postural response is shown by the black boxes on the left side of the scheme; the postural alignment system responsible for the late component is shown as white boxes on the right of the scheme.

For the postural alignment system, rotating visual fields may exert influence on internal estimates of gravity direction by interacting with processing of vestibular information from the semicircular canals (Laurens & Angelaki, 2011). Accurate rotation information, especially from the semicircular canals, is required to extract the gravitational component from the gravito‐inertial signal provided in part by the otoliths, a process that utilises the brainstem and cerebellum (Shaikh *et al*. 2005). Neuronal assemblies in the posterior insular and retroinsular cortex have also been implicated as key sites for integrating visual information with internal estimates of gravity (Indovina *et al*. 2005). Some of these structures may therefore have a role to play in the generation of the later postural response. The regions involved in the early response are uncertain, but nonetheless it seems they have sufficient computational power to help solve the difficult problem of disambiguating environment motion from self‐motion.

## Conclusions

The experiments show that a discrete rotation of the visual field in the frontal plane induces a series of postural adjustments in standing human subjects. The postural adjustments can be reduced to two broad responses with diametrically opposite relationships to changes in the visual‐field velocity profile. This suggests that they serve different functions and are controlled by different neural processes, both of which are biased by rotation of the visual field. We propose that the initial response is generated by the balance control system attempting to minimise unintended body motion, while the later response arises from the postural control system attempting to keep the body's long axis approximately aligned with gravity.

## Additional information

### Competing interests

The authors declare no competing interests.

### Author contributions

The experiments were performed in the Whole‐Body Sensorimotor Control laboratory in UCL Institute of Neurology. B.L.D. conceived and designed the experiments. T.M., J.O. and I.D.G. acquired the data. All authors contributed to data analysis. B.L.D. and T.M. drafted the manuscript. All authors critically revised the manuscript. All authors approved the final version of the manuscript and agree to be accountable for all aspects of the work in ensuring that questions related to the accuracy or integrity of any part of the work are appropriately investigated and resolved. All persons designated as authors qualify for authorship, and all those who qualify for authorship are listed.

### Funding

The work received funding from The Medical Research Council (grant MR/J013234/1). T.M. was supported by a scholarship from the Wolfson Foundation.

**Translational Perspective**

We have found that dynamic visual stimuli produced by rotating the visual field in the frontal plane can induce postural responses in standing human beings through two distinct processes. We hypothesise that one involves the balance control system, which acts rapidly to minimise unintended body movement, while the other involves the posture control system, which acts to orientate the body with respect to gravity. It is probable that these different functions are served by different neural circuitry and that damage to either system could lead to postural instability and falls, but through different pathophysiological mechanisms. Our experimental approach may therefore open a window into understanding better the pathophysiology of postural instability and falls arising from different diseases of the human central nervous system, such as Parkinson's disease and cerebellar disease, or from the neurodegeneration associated with ageing.
